# Mitochondrial mass and DNA repair in breast cancer stem cells

**DOI:** 10.18632/oncotarget.6305

**Published:** 2015-11-11

**Authors:** Richard G. Pestell, Alesandro Fatatis, Xuanmao Jiao

**Affiliations:** Department of Cancer Biology and Sidney Kimmel Cancer Center, Thomas Jefferson University, Philadelphia, PA, USA

**Keywords:** mitochondria, cancer stem cells, cancer metabolism, OXPHOS

Two recent studies by Lisanti and co-workers provide new insights into the importance of mitochondria and the cancer stem cell and its resistance to therapy. Lisanti demonstrated that WNT1/FGF3 enhances breast cancer stem cell (BCSC) expansion via a paracrine loop associated with the induction of mitochondrial biogenesis [1]. Furthermore, Lisanti showed that mitochondria are enriched within BCSC, and that mitochondrial enriched human BCSC are resistant to DNA damage [2]. These finding are consistent with the prior findings that stem cells are resistant to current therapies due to increased DNA repair capacity. The findings by Lisanti are also consistent with prior studies in which an RNAi screen identified Wnt as an inducer of mitochondrial biogenesis [3]. Importantly these studies add to the growing understanding that, in contrast with the reduction in mitochondrial mass in normal stem cells, mitochondrial protein abundance is increased in cancer stem cells.

The studies by Lisanti provide important evidence for a direct link between cancer stem cells and altered mitochondrial metabolism within a heterogeneous breast tumor population. CSCs preferentially perform oxidative phosphorylation over glycolysis compared to non-CSCs and several mechanisms have been described. Firstly, oncogenes, including c-Myc, are sufficient for the induction of OXPHOS and the induction of CSCs [4, 5]. Secondly, metabolic genes are mutated in CSCs in different cancer types. Those mutations in metabolic enzymes may cause gain of function or loss of function. The normal function of isocitrate dehydrogenase-1 (IDH1) and IDH2 is to metabolize isocitrate and NADP+ to yield α-ketoglutarate (αKG) and NADPH [6], [7]. Mutations in IDH1 and IDH2 have recently been identified in a number of different tumor types including prostate cancer. These alterations are gain of function mutations because they drive the synthesis of the ‘oncometabolite’ R-2-hydroxyglutarate (2HG). 2HG-producing IDH mutants prevent the histone demethylation that is required for lineage specific progenitor cells to differentiate into terminally differentiated cells.

Understanding the mechanisms by which increased mitochondrial mass contributes to increased DNA repair is important, as a substantial number of cancer therapies activate DNA damage. How might DNA repair be increased in the CSCs? Firstly, the expression of DNA repair genes are increased in CSCs. Secondly, the induction of the metabolic substrate fumarate, which inhibits JKDM2B histone demethylase activity, may contribute to enhanced DNA damage resistance. Epigenetic silencing of FBP1, which occurs during EMT, decreases ROS to promote the CSC phenotype. An increased reliance on glucose metabolism following FBP1 silencing lowers ROS levels by two mechanisms: decreased mitochondrial respiration and increased NADPH synthesis through pentose phosphate metabolism. In the current studies a shift towards cytosolic glycolysis in the CTC was accompanied by a reduction in ROS. Lower ROS levels promote EMT and CSC phenotype. The reduction in ROS within the CSC population is essential for survival of CSC. Not all studies show oncogenic transformation reduces ROS production, for example, when the entire population of transformed cells was examined, Wnt increased ROS generation and increased DNA damage in the C2C12 muscle cell line [3]. It is likely that subfractionation of mitochondrial high vs mitochondrial low cells within the heterogeneous tumors is necessary to define the stem cell population with reduced ROS and increased DNA repair.

The functional interactions between cancer metabolism and the induction of CSCs are of importance because of the potential to target such metabolic enzymes for cancer therapy. No doubt a dynamic process including mitophagy, movement and thereby apportioning of aged mitochondria into daughter cells [8] as well as local ROS production, kinase signaling such as p38 MAPK, local heterotypic signals from the tissue microenvironment together with intracellular metabolic changes contribute to the CSC phenotype. What potential mechanism repress FBP1 in breast CSC ? As Snail is increased in luminal and basal breast cancer, perhaps the Snail/G9a mediated repression of FBP1 which reduces ROS, may thereby promoting self-renewal potential of CSCs which are exquisitely sensitive to the level of ROS. Given the increased biogenesis of the mitochondria of tumor initiating cancer stem cells and reduced biogenesis of normal stem cells future studies may provide important insights into the distinct mechanisms governing these key cell types.
